# Intradermal Microdroplet Injection of Standard-Diluted Incobotulinumtoxin A for the Treatment of Erythematotelangiectatic Rosacea: A Study From a Dermatology Center in Medellín, Colombia

**DOI:** 10.7759/cureus.78957

**Published:** 2025-02-13

**Authors:** Valentina Dicker, Maria del Mar Serna Posada, Estefania Galeano Piedrahita, Sara Vasquez Villegas, Camila Perez Madrid

**Affiliations:** 1 Aesthetic Medicine, Dra Skin, Medellín, COL; 2 Dermatology, Dra Skin, Medellín, COL

**Keywords:** botulinum toxin, incobotulinum toxin a, redness, rosacea, skin quality

## Abstract

Background: Most previous studies of botulinum toxin A (BoNT/A) use for rosacea have reported using hyperdiluted BoNT/A. Different dilutions than the standard practiced dilution for wrinkle treatment result in confusion, especially if there is more than one injector in an office because daily logistics become complicated.

Objective: To evaluate the safety and efficacy of the use of intradermal injections of incobotulinumtoxin A (INCO) in the standard dilution used for wrinkle treatment (100U vial in 2cc) for the treatment of erythematotelangiectatic rosacea (ETR).

Methods: Data from 13 patients with ETR treated with INCO were prospectively collected in this single-center, open-label preliminary study. Patients answered the Rosacea-specific Quality-of-Life instrument (RosaQol) questionnaire, and a non-treating blinded dermatologist evaluated the Clinician Erythema Assessment (CEA) score using a five-point grading scale at baseline and 15-day and three-month follow-ups.

Results: A significant decrease occurred in the total RosaQoL score at 15-day and three-month (p<0.001) follow-ups compared to baseline. Baseline CEA score was considered moderate (84.6%) or severe (15.4%). Most patients were assessed as almost clear or mild at the 15-day and three-month follow-ups (99.3% and 88.9%, respectively).

Conclusion: Standard 2mL dilution INCO, used for aesthetic indications, may be a safe and effective option for treating ETR-related erythema and flushing.

## Introduction

Rosacea is a chronic inflammatory cutaneous disease with vascular symptoms, such as facial erythema and flushing, that cause major discomfort among patients and may impact social, professional, and family life [1]. Even though new topical drugs have been released for the treatment of rosacea (e.g., brimonidine), the response of erythematotelangiectatic rosacea (ETR) to conventional treatment remains unsatisfactory [2].

The effects of botulinum toxin A (BoNT/A) enable the muscles’ chemical denervation neurologic modulation in sweat and sebaceous glands along with erythema [3]. Thus, in addition to the well-known aesthetic use of BoNT/A in dermatology, it has also been used for rosacea and improvement of skin quality. For ETR treatment, different formulations of BoNT/A and treatment plans have been described; however, most studies have reported the use of hyperdiluted BoNT/A, mainly due to the risk of inadvertent paralysis of underlying skeletal muscles in the treated area [4]. In a pilot study with onabotulinum toxin A (Meditoxin; Meditox, Seoul, Korea) for the treatment of recalcitrant and persistent ETR, three patients complained of unnatural facial expressions and elected to discontinue participation [5]. Moreover, there is no consensus regarding BoNT/A dilution, with volumes of up to 7mL of saline described in the literature [6,7]. Different dilutions for BoNT/A, other than the standard practiced dilution for dynamic wrinkles, may result in confusion, especially if there is more than one injector in an office, because daily logistics become complicated (e.g., different volumes of diluent and more than one type of syringe to administer the BoNT/A are needed) and the risk of dosage errors increases (i.e., number of units/mL) [8]. In this pilot study, we sought to evaluate the safety and efficacy of the use of intradermal injections of incobotulinumtoxin A (INCO) for the treatment of ETR using the standard dilution for wrinkle treatment (100U vial in 2cc).

## Materials and methods

Ethics approval

The study was approved by a centralized institutional review board (approval Acta CEI-141, July 30, 2024). Written informed consent has been provided by all the patients to have the case details and any accompanying images published.

In this preliminary single-center, open-label, evaluator-blinded study, patients with ETR were treated with INCO (Xeomin®; Merz Pharmaceuticals GmbH & Co. KGaA, Frankfurt, Germany). The data included in the study referred to patients treated from August 1 to November 1, 2024, in a dermatological center located in Medellín, Colombia.

Eligible patients were over 18 years of age at the time of enrollment, with a confirmed diagnosis of moderate to severe ETR, as determined by the Clinician Erythema Assessment (CEA), who agreed to adhere to the study protocol. Exclusion criteria included other causes of facial erythema such as lupus erythematous, seborrheic dermatitis, and other causes of facial redness; current use of isotretinoin; or previous treatment with chemical peeling or intense pulsed light in the last six months. No adjuvant treatment for rosacea was received three months before the intervention. None of the subjects were previously treated with BoNT/A for skin quality or for other indications (medically or aesthetically).

Images were obtained at baseline, after a follow-up interval of 15 days, and three months after the procedure. Two-dimensional photographs were taken with a digital camera (Vectra Software, Canfield, NJ, USA) with standardized parameters.

Quality of life assessment

Patients answered the Rosacea-specific Quality-of-Life instrument (RosaQol) questionnaire before the procedure then again 15 days and three months after. RosaQoL is a disease-specific instrument comprising 20 rosacea-related items, with a maximum score of 20, subdivided into three domains (emotion, symptom, and behavior) [1].

CEA score

Facial erythema was evaluated by a non-treating blinded dermatologist using a five-point grading scale of facial erythema severity at baseline and 15 days and three months after treatment [9,10].

Statistical analysis

A descriptive analysis was performed in which categorical variables were summarized by the number (n), percentage (%), and non-categorical variables including mean, standard deviation, median (P50%), and minimum and maximum values.

The behavior of the RosaQoL score across time points (baseline, 15 days, and three months) was evaluated by analysis of variance (ANOVA) with repeated measures, and post-hoc tests were done using the Bonferroni test. CEA's classification data were compared using a binomial test.

Statistical analysis was performed using Stata/SE 15.1 (Stata-Corp, 2017, College Station, TX: StataCorp LLC). All the tests were bi-caudal and were significant if p-value<0.05.

Procedure

**Figure 1:**
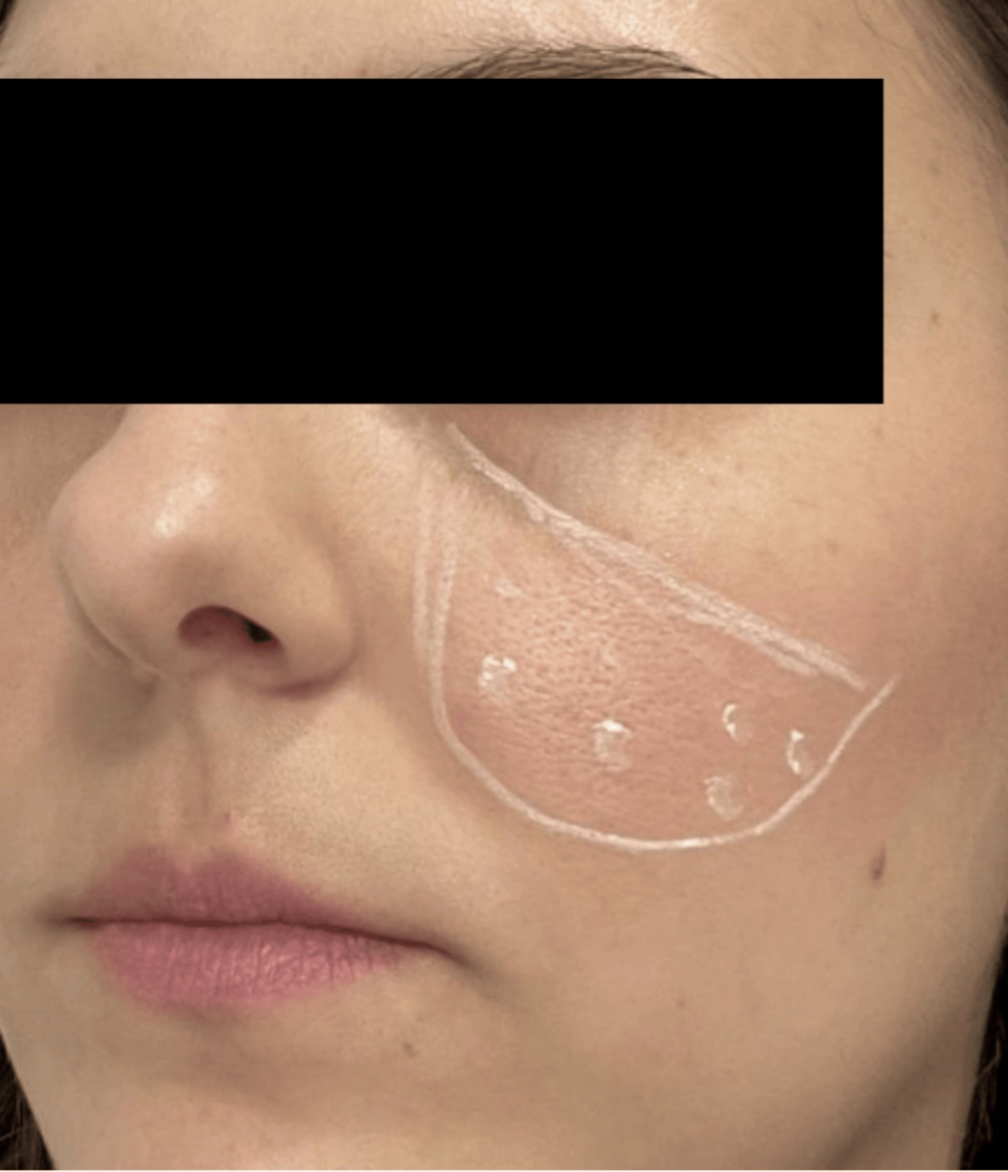
INCO application guide. INCO: incobotulinumtoxin A Written informed consent has been provided by the patient to have the case details and any accompanying images published.

One 100U vial of INCO was diluted in 2 milliliters (mL) of bacteriostatic saline (5U/0.1mL). After images were obtained, erythematous areas to be treated on the cheeks were chosen in each patient and marked (Figure 1).

One unit of INCO (dilution of 1 vial with 2mL of saline) was applied every 0.5-1cm on each cheek where erythema was present, on average 15-20 units per patient for both sides of the face. A 31-gauge (31G) needle was used for intradermal injection. Thereafter, skin disinfection was performed, and without applying anesthetic cream, INCO was injected intradermally (1U/point) at a 0.5-1cm interval between the injection points within the selected area.

## Results

Overall, 10 females (age range: 24-38 years, median age 32 years) and three males (age range: 19-80 years, median 31 years) were injected with a median total of 20U INCO for both sides of the face. Other demographic characteristics are described in Table 1.

**Table 1:** Demographic and basic clinical data of the included patients.

Categorical Variables	Baseline n(%)	15 days	3 months
Total participants	13 (100)	13 (100)	13 (100)
Male	3 (23.1)	3 (23.1)	3 (23.1)
Female	10 (76.9)	10 (76.9)	10 (76.9)
Indoor occupational exposure	12 (92.31)	12 (92.31)	12 (92.31)
Outdoor occupational exposure	1 (7.69)	1 (7.69)	1 (7.69)
Non-categorical Variables	Mean ± SD	Range	Median (P50)
Age (years)	35.53 ± 13.89	24-80	32

**Figure 2:**
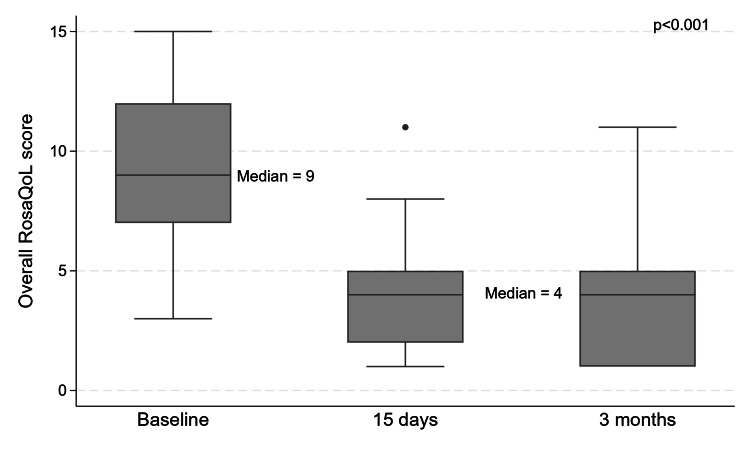
Box plot of RosaQoL answers at each time point. Note: There was a decrease in the median score at the 15-day and three-month follow-ups. RosaQoL: Rosacea-specific Quality-of-Life instrument

**Figure 3:**
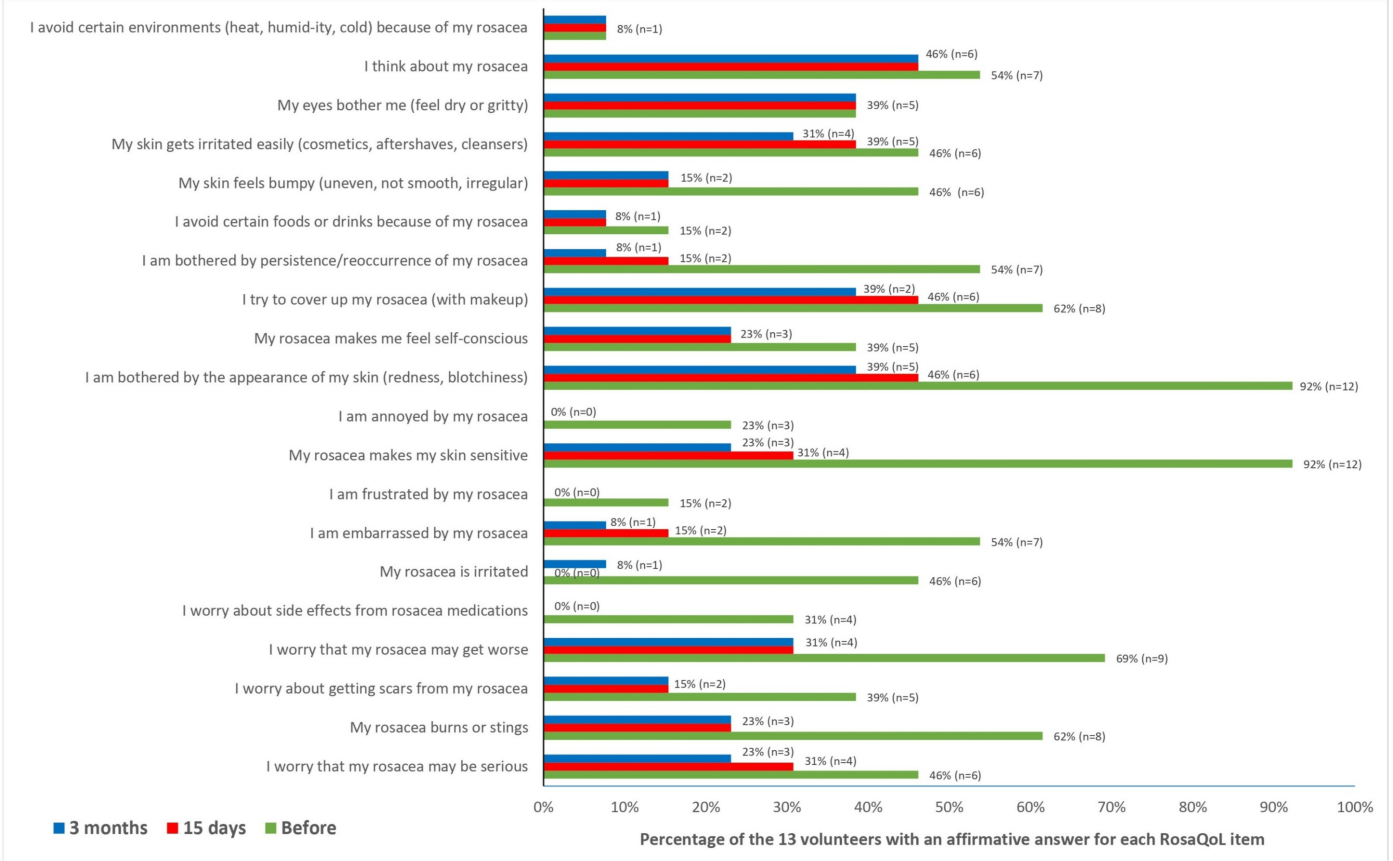
Distribution of affirmative answers to each RosaQoL question at each time point. RosaQoL: Rosacea-specific Quality-of-Life instrument

The mean baseline RosaQoL score was 9.3±3.6 points, while on day 15, it was 4.0±3.0 points, which was maintained until the end of the follow-ups at three months (Figure 2). Apart from two questions related to eye symptoms and behavior (“My eyes bother me (feel dry or gritty)” and “I avoid certain environments (heat, humid­ity, cold) because of my rosacea”), most responses presented a decrease in the number of affirmative answers after treatment, meaning improvement in their overall symptoms (Figure 3).

For all patients, at the 15-day and three-month follow-ups, no positive responses for questions 5 (“I worry about side effects from rosacea medications”), 8 (“I am frustrated by my rosacea”), and 10 (“I am annoyed by my rosacea”) were observed. There was a significant decrease of 5±0.8 points (p<0.001) at 15 days and 5.5±0.8 points (p<0.001) at the three-month evaluation in the total RosaQoL score when compared to baseline (Figure 3).

**Figure 4:**
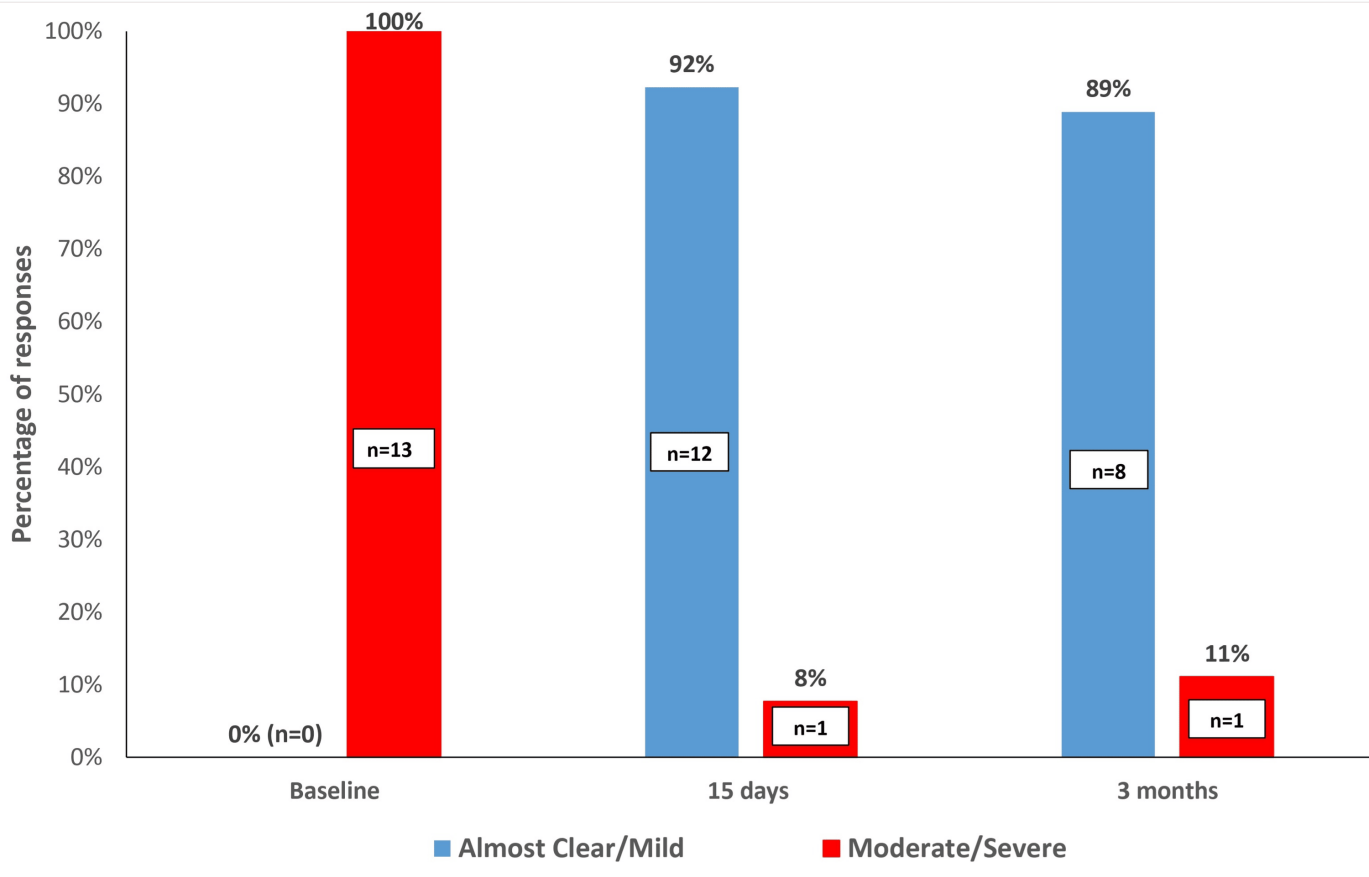
Clinician Erythema Assessment (CEA) score at each time point.

Nonetheless, no significant difference was observed comparing the 15-day and three-month evaluations (p=0.705). At baseline, the CEA score was considered moderate (84.6%) or severe (15.4%) (Figure 4).

Severe and moderate, and almost clear and mild, were grouped. Improvement in the value of the CEA was observed at the 15-day and three-month follow-ups. A significant percentage of patients were deemed “almost clear or mild” compared with baseline and at the 15-day (p=0.003) and three-month (p=0.039) follow-ups.

Most of the patients were assessed as almost clear or mild at the 15-day and three-month follow-ups (99.3% (95% CI: 64.0%; 99.8%) and 88.9% (95% CI: 51.8%; 99.7%) respectively), and none were deemed as severe. Data are presented in Table 2.

**Table 2:** Distribution of CEA score as assessed by investigator at each time point. CEA: Clinician Erythema Assessment

CEA	Before (n=13)	15 days (n=13)	3 months (n=9)
Almost clear	0 (0.0%)	8 (61.5%)	6 (66.7%)
Mild	0 (0.0%)	4 (30.8%)	2 (22.2%)
Moderate	11 (84.6%)	1 (7.7%)	1 (11.1%)
Severe	2 (15.4%)	0 (0.0%)	0 (0.0%)

The CEA score was not evaluated at three months for four patients because the patients were out of town but answered the RosaQoL questionnaire remotely.

**Figure 5:**
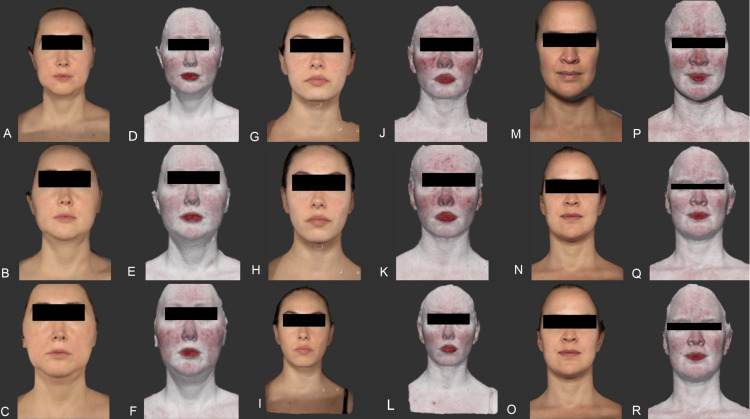
Patients before and after the application of INCO. Left panel: A 36-year-old female; baseline pictures (A, D), 15-day assessment (B, E), and 3-month (C, F) follow-ups. Middle panel: A 33-year-old female; baseline pictures (G, J), 15-day assessment (H, K), and 3-month (I, L) follow-ups. Right panel: A 38-year-old female; baseline pictures (M, P), 15-day assessment (N, Q), and 3-month (O, R) follow-ups. In the pictures taken with Vectra (D, E, F, J, K, L, P, Q, R), the improvement of the erythema is more evident compared to clinical pictures. INCO: incobotulinumtoxin A Written informed consent has been provided by the patients to have the case details and any accompanying images published.

All patients presented only mild and self-limited adverse events during the procedure described as low pain during injection using Visual Analogue Scale, and mild transitory erythema that lasted no more than three to five minutes after the treatment was finished (Figure 5). 

## Discussion

The precise pathogenesis of facial erythema in rosacea is unknown, and the interplay among factors that have been cited in the literature (e.g., vasodilation, neurogenic inflammation, immune system dysregulation, upregulation of metalloproteinases) remain to be elucidated [7]. One theory is that repeated episodes of vasodilation would result in loss of vascular tone and permanent vessel dilation, leading to the release and delayed clearing of inflammatory mediators [9,11]. Activated mast cells seem upregulated in rosacea, along with the release of inflammatory and vasoactive mediators. Moreover, skin blood flow is increased through the sympathetic cholinergic nerves in addition to the release of acetylcholine. Other mediators, such as vasoactive intestinal peptides and pituitary adenylate cyclase-activating peptides in the skin, have been reported to be active transmitters involved in vasodilation [7,12].

Currently, oral and topical medications, as well as lasers, are routinely prescribed for rosacea. Despite the improvement of erythema, they often fail to control facial flushing [13] through the blockage of vasodilatory neuropeptides release (e.g., acetylcholine and vasoactive intestinal peptide) and modulation of other neuropeptides thought to be involved in the pathogenesis of rosacea (e.g., vascular endothelial growth factor, P substance, and calcitonin gene-related peptide). BoNT/A has been shown to influence vasodilation, the release of inflammatory mediators, and mast cell degranulation, thereby controlling rosacea’s neurovascular symptoms such as erythema and flushing [3,14]. Furthermore, murine studies demonstrate that BoNT/A significantly decreases mast cell counts and cathelicidin LL-37-induced skin erythema [15].

In a recent systematic review and meta-analysis of BoNT/A use for rosacea, 22 studies were included, and varying degrees of improvement in signs and symptoms were reported, along with reduced CEA scores and mild and self-limiting adverse events [4]. In the 12 studies available in the English language in which BoNT/A has been used as a single treatment for rosacea (not combined with other treatments), a concentration of BoNT/A lower than the usual used for wrinkles (hyperdiluted BoNT/A) was reported in most cases, mainly due to the risk of inadvertent paralysis of underlying skeletal muscles in the treated area. Despite the improvement observed in all latter studies, it is important to consider the daily logistics of using different dilutions (different volumes of saline) for rosacea because they may result in confusion and risk of dosage errors, especially when there is more than one injector in an office. In this preliminary study, we injected 13 patients with the dilution of INCO for wrinkles (100U in 2mL of saline), and the patients presented only mild and self-limiting adverse events, suggesting that injection of a regular concentration of INCO should be safe. While choosing the BoNT/A formulation, it is particularly relevant to consider the latter’s spread, especially when aiming to minimize the risk of undesirable toxin diffusion into nearby untargeted muscles. It has been shown that onabotulinum toxin A and INCO A have comparable spread, whereas abobotulinumtoxin A has a significantly greater spread than INCO [16].

Even though there is no consensus on the number of units to treat rosacea, we kept the total dose per side under 15U of INCO to avoid the risk of undesirable side effects in the underlying musculature. Despite the total lower dose compared to some of the previous reports [7,14], there did not seem to be any compromise in efficacy and improvement of flushing symptoms, as corroborated by the significant improvement in the total RosaQoL scores at the 15-day and three-month assessments, as well as improvement in CEA score [10] as assessed by the investigator (Figures 2-5).

The limitations of this study largely relate to its small population along with similar demographic characteristics, such as geographic location, which may limit the generalizability of the findings to a broader population. These exploratory studies require follow-up using comparative designs conducted with a larger patient cohort for increased power. Additionally, using a convenience sampling method to select participants makes a selection bias possible. Furthermore, the three-month study duration and follow-up may not adequately capture long-term outcomes.

## Conclusions

Given the limited efficacy of conventional treatments of ETR-related erythema and flushing, using a standard 2mL dilution of INCO, traditionally employed for aesthetic purposes, may be a safe and effective alternative. This approach has shown improvements in quality of life and erythema severity in this study, as evidenced by lower scores in the RosaQoL questionnaire and standardized clinical scoring systems such as the CEA. This study addresses the lack of consensus regarding the optimal BoNT/A dilution in literature and underscores the need for further research to establish standardized protocols.

## References

[1] Zhang H, Tang K, Wang Y, Fang R, Sun Q (2021). Use of botulinum toxin in treating rosacea: a systematic review. Clin Cosmet Investig Dermatol.

[2] Luque A, Rojas AP, Ortiz-Florez A, Perez-Bernal J (2021). Botulinum toxin: an effective treatment for flushing and persistent erythema in rosacea. J Clin Aesthet Dermatol.

[3] Sayed KS, Hegazy R, Gawdat HI (2021). The efficacy of intradermal injections of botulinum toxin in the management of enlarged facial pores and seborrhea: a split face-controlled study. J Dermatolog Treat.

[4] He G, Yang Q, Wu J, Huang Y, Zheng H, Cheng H (2024). Treating rosacea with botulism toxin: protocol for a systematic review and meta-analysis. J Cosmet Dermatol.

[5] Park KY, Kwon HJ, Kim JM, Jeong GJ, Kim BJ, Seo SJ, Kim MN (2018). A pilot study to evaluate the efficacy and safety of treatment with botulinum toxin in patients with recalcitrant and persistent erythematotelangiectatic rosacea. Ann Dermatol.

[6] Dayan SH, Pritzker RN, Arkins JP (2012). A new treatment regimen for rosacea: onabotulinumtoxinA. J Drugs Dermatol.

[7] Yang R, Liu C, Liu W, Luo J, Cheng S, Mu X (2022). Botulinum toxin A alleviates persistent erythema and Flushing in patients with erythema telangiectasia rosacea. Dermatol Ther (Heidelb).

[8] Bass Kaplan J (2016). The dilution confusion: easy dosing for botulinum toxins. Plast Surg Nurs.

[9] Nicholson K, Abramova L, Chren MM, Yeung J, Chon SY, Chen SC (2007). A pilot quality-of-life instrument for acne rosacea. J Am Acad Dermatol.

[10] Tan J, Liu H, Leyden JJ, Leoni MJ (2014). Reliability of clinician erythema assessment grading scale. J Am Acad Dermatol.

[11] Hanna E, Xing L, Taylor JH, Bertucci V (2022). Role of botulinum toxin A in improving facial erythema and skin quality. Arch Dermatol Res.

[12] Rodrigues-Braz D, Zhao M, Yesilirmak N, Aractingi S, Behar-Cohen F, Bourges JL (2021). Cutaneous and ocular rosacea: common and specific physiopathogenic mechanisms and study models. Mol Vis.

[13] Kerscher M, Roll S, Becker A, Wigger-Alberti W (2012). Comparison of the spread of three botulinum toxin type A preparations. Arch Dermatol Res.

[14] Kim YS, Hong ES, Kim HS (2017). Botulinum toxin in the field of dermatology: novel indications. Toxins (Basel).

[15] Calvisi L, Diaspro A, Sito G (2022). Microbotox: a prospective evaluation of dermatological improvement in patients with mild-to-moderate acne and erythematotelangiectatic rosacea. J Cosmet Dermatol.

[16] Choi JE, Werbel T, Wang Z, Wu CC, Yaksh TL, Di Nardo A (2019). Botulinum toxin blocks mast cells and prevents rosacea like inflammation. J Dermatol Sci.

